# Temperature-Dependent Irradiation-History Effects in Tungsten Under Sequential Helium and Hydrogen Plasma Exposure

**DOI:** 10.3390/ma19194038

**Published:** 2026-09-22

**Authors:** Zarina Satbayeva, Bauyrzhan Rakhadilov, Yerasyl Naimankumaruly, Yernar Turabekov, Yelaman Batanov

**Affiliations:** 1 “PlasmaScience” LLP, Ust-Kamenogorsk 070000, Kazakhstan; zarinavkgu@gmail.com (Z.S.); rakhadilovb@gmail.com (B.R.);; 2 Department of Technical Physics and Thermal Power Engineering, Faculty of Engineering and Technology, Shakarim University, Semey 071400, Kazakhstan; 3 International School of Engineering, D. Serikbayev East Kazakhstan Technical University, Ust-Kamenogorsk 070010, Kazakhstan

**Keywords:** tungsten, helium pre-irradiation, hydrogen desorption, sequential plasma exposure, irradiation history, plasma–surface interaction, thermal desorption spectroscopy, nanomechanical properties

## Abstract

Tungsten plasma-facing components in fusion devices may experience sequential exposure to helium and hydrogen, making irradiation history an important factor in their subsequent structural and hydrogen-release behavior. This study investigated polycrystalline tungsten exposed to He-only, H_2_-only, and sequential He → H_2_ plasmas at 700, 900, 1100, and 1300 °C. Surface and cross-sectional scanning electron microscopy, X-ray diffraction, instrumented indentation, and thermal desorption spectroscopy (TDS) were used to characterize the resulting changes. SEM observations showed that the largest differences among the H_2_-only, He-only, and sequential He → H_2_ exposure conditions occurred at 1100 °C, whereas predominantly grain-scale surface morphology was observed for all three conditions at 1300 °C. The bcc-W structure was retained under all investigated conditions, with no detectable secondary crystalline phases. The apparent coherent diffraction domain size, estimated from the W(110) reflection using the Scherrer equation, increased monotonically with irradiation temperature, from approximately 48–55 nm at 700 °C to approximately 126 nm at 1300 °C. At 1100 °C, the H_2_-only specimen exhibited a larger apparent coherent diffraction domain size than the He-containing specimens. The nanomechanical response also depended on temperature and exposure history. TDS revealed distinct high-temperature hydrogen-desorption behavior for the sequential He → H_2_ specimens, particularly at 1100 and 1300 °C. Overall, the results indicate that the tungsten response under the present sequential protocol depends on both temperature and exposure history, and that structural convergence at elevated temperature does not necessarily correspond to equivalent hydrogen-release behavior.

## 1. Introduction

Tungsten (W) is widely regarded as one of the principal plasma-facing materials for ITER and future fusion devices because of its exceptionally high melting temperature, high thermal conductivity, low sputtering yield, and favorable resistance to plasma erosion [1,2,3,4]. Nevertheless, plasma-facing tungsten will operate under a complex combination of high steady-state and transient heat loads, intense particle fluxes, hydrogen isotopes originating from the fuel cycle, helium produced as fusion ash, and eventually neutron-induced displacement damage [4,5,6]. These conditions can substantially modify the defect population, surface morphology, gas-retention behavior, and mechanical properties of tungsten, thereby affecting both component lifetime and the hydrogen-isotope inventory.

Hydrogen isotopes are highly mobile in an undamaged tungsten lattice but can be strongly trapped at vacancies, vacancy clusters, dislocations, grain boundaries, voids, and other intrinsic or irradiation-induced defects [7,8,9]. Low-energy, high-flux hydrogen-isotope plasma exposure can therefore produce substantial subsurface accumulation, blistering, cracking, and changes in surface morphology even when the incident ion energy is below the threshold for displacement damage [7,8]. Hydrogen retention is also strongly temperature-dependent because diffusion, detrapping, defect recovery, and the population of available trapping sites evolve simultaneously with temperature [8]. Atomistic calculations further show that vacancy-type defects provide energetically favorable trapping configurations for multiple hydrogen atoms [9].

Helium produces a qualitatively different response because of its extremely low solubility in tungsten and strong interaction with vacancy-type defects. Low-energy, high-flux He plasma exposure can result in the formation of He–vacancy complexes, nanobubbles, cavities, and, under appropriate combinations of temperature, ion flux, and fluence, highly developed nanostructured or fuzz-like surfaces [10,11,12,13]. The morphology and connectivity of these defects depend strongly on temperature, since increasing defect mobility promotes bubble growth, coalescence, surface restructuring, and the development of open-volume defects. Consequently, a He-modified near-surface region is not merely a damaged layer but a defect structure capable of altering subsequent particle transport and trapping.

The interaction between hydrogen and helium in tungsten is therefore not necessarily additive. Simultaneous or mixed H/D–He irradiation studies have shown that the presence of He can either suppress or enhance hydrogen-isotope retention depending on irradiation temperature, ion fluence, He concentration, and the resulting defect morphology [13,14,15,16]. Helium-seeded deuterium plasma has been reported to reduce D retention under some conditions by altering the population of available trapping sites [14], whereas He-induced bubbles, vacancy complexes, and dislocation structures may provide additional trapping regions under other conditions [13,15,16]. These apparently different responses emphasize that H–He interaction is controlled not simply by the amounts of implanted gas but by the irradiation-induced microstructure in which transport and trapping occur.

The sequence of particle exposure introduces an additional variable. During simultaneous irradiation, hydrogen- and helium-related defects develop within a common evolving microstructure, whereas sequential exposure allows a He-modified layer to form before hydrogen implantation begins. Nishijima et al. demonstrated that He plasma pre-exposure can either decrease or markedly increase subsequent deuterium retention depending on the He exposure temperature [17]. At lower temperatures, occupation of vacancy-type trapping sites by He may reduce the number of sites available to D, whereas high-temperature He exposure can generate bubbles and secondary defects that subsequently act as additional trapping regions. Xia et al. further showed that a pre-existing nanoscale He-bubble layer can initially suppress hydrogen-induced blistering, while prolonged subsequent hydrogen exposure modifies the He-induced structure and enables hydrogen transport through the affected region [18]. More recent sequential He–D experiments similarly indicate that suppression of surface blistering after He pre-exposure does not necessarily correspond to reduced hydrogen-isotope retention [19]. These results demonstrate that surface morphology and retained hydrogen inventory must be evaluated independently.

Helium-induced defect structures can also modify the mechanical and crystalline response of tungsten. Nanoindentation investigations have demonstrated He-induced changes in hardness associated with interactions between implanted He, vacancy defects, and dislocations [20,21]. Plasma-exposure experiments likewise reveal temperature-dependent changes in tungsten microstructure and mechanical properties after He irradiation [22]. Huang et al. recently showed that tungsten subjected to combined He-plasma irradiation and thermal loading retained the bcc-W crystal structure while exhibiting changes in diffraction characteristics, surface damage, and mechanical response [23]. These observations indicate that irradiation-induced defects and thermally activated structural evolution act simultaneously, and their relative contributions may change substantially with temperature.

Temperature is therefore a key parameter linking surface morphology, crystalline structure, mechanical response, and gas retention. At lower temperatures, reduced defect mobility can preserve irradiation-induced defect populations, whereas at elevated temperatures bubble evolution, defect recovery, grain-boundary restructuring, and microstructural coarsening become progressively more important [4,6,22,23,24]. Direct comparisons of hydrogen- and helium-plasma exposure further demonstrate that the resulting surface response depends strongly on both plasma species and exposure temperature [24]. However, systematic experiments comparing He-only, H_2_-only, and sequential He → H_2_ exposure over the same broad temperature interval remain relatively limited. In particular, the transition from irradiation-history-dominated behavior to thermally dominated structural evolution has not been sufficiently clarified.

In our previous work, polycrystalline tungsten was exposed to steady-state mixed He/H_2_ plasma at 1100 °C while the He fraction was varied from 5% to 50% [25]. Increasing the He fraction produced progressively stronger modification of the near-surface morphology and altered both the nanomechanical response and hydrogen thermal-desorption behavior. These results demonstrated an association between He-induced structural modification and hydrogen-desorption behavior. However, because He and H_2_ were introduced simultaneously, the contribution of a pre-existing He-modified structure could not be separated from defects generated concurrently during mixed-plasma exposure. The KAZ-PSI linear plasma simulator used in these studies enables controlled steady-state exposure of tungsten to helium and hydrogen plasmas and therefore provides an appropriate platform for investigating sequential irradiation histories [26].

The present study investigates temperature-dependent irradiation-history effects in polycrystalline tungsten subjected to He-only, H_2_-only, and sequential He → H_2_ plasma exposure at 700, 900, 1100, and 1300 °C. The He-only series characterizes the structural state produced by helium exposure, whereas the H_2_-only series provides the corresponding hydrogen-plasma reference. The sequential He → H_2_ condition is used to evaluate how a pre-existing He-modified near-surface structure influences the subsequent response to hydrogen plasma. Surface and cross-sectional scanning electron microscopy, X-ray diffraction, instrumented indentation, and thermal desorption spectroscopy are combined to correlate irradiation-history-dependent changes in morphology and crystalline structure with the nanomechanical and hydrogen-desorption responses. The principal objective is to determine how the influence of He pre-exposure evolves with temperature and whether irradiation-history effects persist or become progressively dominated by thermally activated structural evolution at elevated temperatures.

## 2. Materials and Methods

Commercially pure, pre-annealed polycrystalline tungsten (W), manufactured by Plansee SE (Reutte, Austria), was used in this study. The nominal purity of the material was ≥99.97 wt.%, and the density was greater than 19.1 g/cm^3^. Cylindrical specimens with a diameter of 6 mm and a height of 5 mm were prepared from the same tungsten batch. After surface preparation, the nominal specimen dimensions remained approximately 6 mm in diameter and 5 mm in height, because only a small amount of material was removed during grinding and polishing.

The plasma-facing surfaces were mechanically ground using SiC abrasive papers up to P2500 grit and subsequently polished with a 1 μm diamond suspension. After polishing, the specimens were ultrasonically cleaned in acetone for 5 min and then in isopropanol for 5 min. The specimens were dried before plasma exposure.

Plasma irradiation was performed using the KAZ-PSI linear plasma simulator developed by PlasmaScience LLP (Ust-Kamenogorsk, Kazakhstan) for plasma–surface interaction studies [26]. Before each experiment, the interaction chamber was evacuated to a base pressure in the range of 10^−5^–10^−4^ Pa. During plasma exposure, the working pressure was approximately 1 Pa. The working pressure was maintained close to 1 Pa using a gas flow rate of approximately 50 sccm for both He and H_2_ and by partially throttling the turbomolecular pump with a two-position pneumatic valve.

A total of 12 tungsten specimens were irradiated. Three plasma-exposure conditions were investigated at nominal surface temperatures of 700, 900, 1100, and 1300 °C: (i) He-only exposure, (ii) H_2_-only exposure, and (iii) sequential He → H_2_ exposure. Due to the duration and experimental constraints of the sequential plasma-exposure protocol, one independently irradiated specimen was used for each combination of exposure condition and temperature. To reduce uncontrolled variability, all specimens were prepared from the same tungsten batch and subjected to identical surface-preparation procedures. For the nanoindentation measurements, multiple local measurements were performed on each specimen to characterize within-specimen spatial variability. The selected temperature range was chosen to cover a broad regime in which defect mobility, gas transport, and thermally activated structural evolution in tungsten change substantially. A 200 °C interval was used to provide a systematic comparison of the temperature-dependent response. The temperature of 1100 °C was additionally included to enable comparison with our previous mixed He/H_2_ plasma study [25].

The He-only and H_2_-only specimens were exposed to the corresponding plasma for 1 h. For the sequential He → H_2_ condition, the specimens were first irradiated with He plasma for 1 h and then immediately exposed to H_2_ plasma for an additional 1 h. During the transition between the He and H_2_ irradiation stages, the vacuum chamber remained closed and the vacuum was not broken. The plasma discharge was briefly interrupted while the gas supply was switched from He to H_2_, without atmospheric exposure. The specimen surface temperature was continuously monitored by the infrared pyrometer, and no appreciable temperature decrease was observed during the short gas-changeover period. After stabilization of the H_2_ flow and working pressure, the H_2_-plasma stage was initiated. No intentional intermediate annealing was performed between the two irradiation stages. The selected nominal surface temperature was maintained during both stages. Thus, the total plasma-exposure duration was 1 h for the He-only and H_2_-only specimens and 2 h for the sequentially irradiated specimens.

Helium and molecular hydrogen were supplied independently during the corresponding irradiation stages. The gas flow rate was approximately 50 sccm for both the He and H_2_ plasma exposures.

The H_2_-only specimens were used as reference conditions for evaluating the subsequent hydrogen-plasma response, while the He-only specimens characterized the state produced by He exposure before the hydrogen stage. Because the sequential He → H_2_ specimens received an additional 1 h of He irradiation, the experiment was not designed as an equal-total-exposure-time or equal-total-fluence comparison. Accordingly, differences between the H_2_-only and He → H_2_ conditions are interpreted in the context of the complete sequential irradiation history, which includes the preceding He-exposure stage.

The ion flux was estimated from Langmuir-probe measurements performed near the target region using the probe integrated into the KAZ-PSI interaction chamber. The probe current–voltage characteristics were used to determine the plasma parameters required for estimating the ion flux. The estimated uncertainty of the ion-flux determination was approximately ±15%. The corresponding fluence was calculated as Φ = Γt, where Γ is the estimated ion flux and t is the irradiation time.

The main plasma exposure conditions are summarized in Table 1.

The plasma-facing surface temperature was monitored using a DIKAI infrared pyrometer (model DIT-6H, DIKAI Optoelectronic Technology Co., Ltd., Shenzhen, China) focused on the central plasma-exposed region of the specimen. The nominal irradiation temperatures were 700, 900, 1100, and 1300 °C, with an estimated measurement uncertainty of ±15 °C.

Surface and transverse fracture morphologies were examined using an SEM3200 scanning electron microscope (CIQTEK Co., Ltd., Hefei, China) equipped with a tungsten cathode. SEM observations were performed at an accelerating voltage of 15 kV under low-vacuum conditions. Surface micrographs used for comparison were acquired from the central plasma-exposed region at the same nominal magnification of ×2000. Transverse fracture surfaces were used for qualitative comparison of the subsurface morphology and were not used for quantitative determination of grain size or modified-layer thickness. The gas composition of individual pore-like or cavity-like features was not determined by SEM.

The crystalline structure of the irradiated tungsten specimens was examined by X-ray diffraction using a DX-27mini diffractometer (Dandong Haoyuan Instrument Co., Ltd., Dandong, China) with Cu Kα radiation (λ = 0.154059 nm). The X-ray tube was operated at 40 kV and 12 mA. Diffraction patterns were recorded over the 2θ range of 30–90° with a step size of 0.02° and a counting time of 1 s per point. The slit configuration was 1°/1°/0.4 mm, and the monochromator was enabled. The instrumental broadening was evaluated using a standard reference specimen measured under the same instrumental conditions and corresponded to an FWHM of approximately 0.05° in 2θ. The diffraction patterns were analyzed using HighScore Plus software (version 3.0e). The position and full width at half maximum (FWHM) of the W(110) reflection were determined by fitting the experimental peak profile with a pseudo-Voigt function. The apparent coherent diffraction domain size D was calculated from the FWHM of the W(110) reflection.

Changes in peak position, FWHM, and relative diffraction intensity were used for comparative evaluation of the irradiated specimens. Relative peak intensities were interpreted qualitatively as possible changes in preferred crystallographic orientation and were not used to determine phase fractions.

The FWHM values were converted from degrees to radians according to

(1) $$\beta =\mathrm{FWHM}\frac{\pi }{180},$$

where β is the peak width expressed in radians.

The apparent coherent diffraction domain size D was estimated from the W(110) reflection using the Scherrer equation:

(2) $$D\;=\;\frac{K\lambda }{\beta cos\theta },$$

where K = 0.9 is the Scherrer shape factor, λ = 0.154059 nm is the wavelength of Cu Kα radiation, β is the FWHM in radians, and θ is the Bragg angle. Although the instrumental broadening was evaluated from the standard-reference measurement, it was not subtracted from the fitted W(110) peak widths. Therefore, the calculated D values are reported as apparent coherent diffraction domain sizes and are used only for comparative evaluation between irradiation conditions.

Thermal desorption spectroscopy (TDS) was used to investigate hydrogen release from the irradiated tungsten specimens. The TDS system consisted of a high-vacuum chamber, sample holder, tantalum resistance heater, temperature-monitoring system, pumping system, and quadrupole mass spectrometer. The TDS measurements were used for comparative evaluation of the desorption behavior and relative hydrogen-release intensity among the investigated exposure conditions. Because the quadrupole mass-spectrometer signal was not converted to an absolute hydrogen desorption flux, the TDS results are reported on a relative basis rather than as absolute hydrogen inventories.

Before the main TDS measurement, each specimen was heated from room temperature to 100 °C over 15 min and held at 100 °C for 60 min under vacuum. The specimen was then heated from 100 to 1100 °C over 200 min at a nominal heating rate of 5 °C min^−1^, followed by a 30 min isothermal hold at 1100 °C.

Molecular hydrogen was monitored at *m*/*z* = 2. The TDS spectra were evaluated using the position of the principal desorption maximum, the shape of the desorption curve, and the high-temperature desorption behavior.

The nanomechanical properties of the irradiated tungsten specimens were measured using a FISCHERSCOPE HM2000 instrument (Helmut Fischer GmbH, Sindelfingen, Germany) in accordance with DIN EN ISO 14577-1 [27]. A four-sided Vickers diamond indenter with a face angle of 136° was used.

The maximum load was 5.000 mN and was applied over 10 s, followed by a 5 s dwell at maximum load. The indenter approach velocity was 2 μm s^−1^. The indentation data were processed using WIN-HCU software (version 7.4).

The measured parameters included Martens hardness HM, indentation hardness HIT, converted Vickers hardness HV, and indentation modulus EIT. Fifteen indentations were performed within the central plasma-exposed region of each specimen. Measurements affected by visible pores, cracks, specimen edges, or surface contamination were excluded from the analysis. Mean values and standard deviations were calculated from the valid indentation measurements. Accordingly, the reported standard deviations characterize the local spatial variability of the nanomechanical response within each irradiated specimen and provide a measure of the consistency of the indentation data obtained under a given exposure condition.

## 3. Results and Discussion

Figure 1 compares the surface morphology of tungsten after H_2_-only, He-only, and sequential He → H_2_ plasma exposure at 700, 900, 1100, and 1300 °C. The columns correspond to increasing irradiation temperature, while the rows correspond to the three exposure conditions.

At 700 °C, the surfaces of the H_2_-only, He-only, and He → H_2_ specimens retain the polishing grooves of the initial surface (Figure 1a,e,i). Local depressions and isolated dark-contrast features are present, but the original polishing relief remains clearly distinguishable in all three specimens.

With increasing temperature to 900 °C (Figure 1b,f,j), the polishing grooves remain visible, while the surface relief becomes less uniform. The H_2_-only and He → H_2_ specimens exhibit irregular fine-scale features distributed between the polishing traces, whereas the He-only specimen shows a comparatively more uniform modified surface. No large open cavities are observed at this temperature.

The differences among the exposure conditions become more evident at 1100 °C (Figure 1c,g,k). The H_2_-only specimen exhibits a heterogeneous surface with locally developed irregular relief. The He-only specimen exhibits more rounded depressions and pore-like features than the H_2_-only specimen. The sequential He → H_2_ specimen shows a more developed irregular surface with numerous pore-like and cavity-like features, while the initial polishing relief is largely absent. The gas composition of these features cannot be determined from SEM observations alone.

At 1300 °C (Figure 1d,h,l), the surface morphology is dominated by polygonal grains and clearly defined grain boundaries. Intragranular terrace-like and striated features are visible, particularly in the H_2_-only specimen. Local cavity-like features remain visible in the He-containing specimens. In contrast to the 700–1100 °C specimens, the surface morphology at 1300 °C is primarily characterized by grain-scale features in all three exposure conditions.

Overall, the SEM observations show a transition from polishing-dominated morphology at 700 °C to increasingly irregular and pore-like surface features at 900–1100 °C, followed by grain-dominated morphology at 1300 °C. The largest visual difference between the H_2_-only and sequential He → H_2_ surfaces is observed at 1100 °C, whereas the surface morphologies become more similar at 1300 °C.

Figure 2 presents cross-sectional fracture SEM images of tungsten after H_2_-only, He-only, and sequential He → H_2_ plasma exposure at 700, 900, 1100, and 1300 °C. The fracture surfaces were used for qualitative comparison of the subsurface morphology as a function of irradiation temperature and exposure history.

At 700 °C, the fracture surfaces of the H_2_-only, He-only, and He → H_2_ specimens exhibit a relatively fine and elongated morphology (Figure 2a,e,i). The near-surface region appears compact, and no large cavity-like features are evident in the examined fields.

With increasing temperature to 900 °C (Figure 2b,f,j), elongated and locally columnar features remain visible, while the fracture morphology becomes less uniform. The He → H_2_ specimen exhibits a more irregular fracture relief than at 700 °C, whereas the H_2_-only and He-only specimens retain a predominantly fine elongated morphology.

At 1100 °C (Figure 2c,g,k), the fracture morphology becomes coarser in all three exposure conditions. Larger irregular fracture regions are observed together with local cavity-like features in the He-containing specimens. The sequential He → H_2_ specimen shows the clearest transition from the finer elongated morphology observed at 900 °C to a coarser grain-scale fracture structure.

At 1300 °C (Figure 2d,h,l), the fracture surfaces are dominated by large grain-scale regions. The fine elongated features observed at 700–900 °C are largely absent, and coarse polygonal fracture regions are present in all three exposure conditions. The differences among the H_2_-only, He-only, and He → H_2_ specimens are less pronounced than at 1100 °C.

Overall, the cross-sectional fracture observations show a transition from relatively fine and elongated features at 700–900 °C to a coarser grain-scale morphology at 1100–1300 °C. The most evident change occurs between 900 and 1100 °C. Because the observations were obtained from fracture surfaces rather than polished metallographic cross sections, the images were used qualitatively and were not used to determine the true grain size or modified-layer thickness.

The temperature-dependent morphological evolution observed in Figure 1 and Figure 2 is consistent with previous reports showing that the near-surface response of tungsten to He-containing plasma depends strongly on irradiation temperature and on the defect structure formed during He exposure [17,18,24]. In the present experiments, the largest difference between the H_2_-only and He-containing conditions occurs at 1100 °C, whereas the surface and fracture morphologies become more similar at 1300 °C. This behavior suggests that the influence of the preceding He exposure is most evident within an intermediate temperature range, while grain-scale structural evolution becomes increasingly important at the highest temperature. Accordingly, the observed features are described morphologically as pore-like and cavity-like structures without assigning them to a specific gas species. Detailed identification of their internal structure and size distribution would require complementary high-resolution cross-sectional characterization, such as TEM or FIB-based analysis.

X-ray diffraction was used to compare the crystalline structure of tungsten after H_2_-only, He-only, and sequential He → H_2_ plasma exposure at 700, 900, 1100, and 1300 °C. The diffraction patterns obtained for the three exposure conditions at each temperature are shown in Figure 3.

All diffraction patterns contain the characteristic reflections of body-centered cubic tungsten, corresponding to the (110), (200), (211), and (220) planes. No additional diffraction peaks attributable to secondary crystalline phases were detected within the sensitivity of the XRD measurements.

The relative intensities of the tungsten reflections vary among the irradiation conditions and temperatures. These variations were considered qualitatively because diffraction intensity can be affected by crystallographic texture and the condition of the irradiated surface. Therefore, changes in relative peak intensity were not interpreted as changes in phase concentration.

The position and broadening of the W(110) reflection were used for quantitative comparison of the irradiated specimens. Because small changes in peak position may also be affected by specimen positioning and instrumental factors, the peak shifts were interpreted cautiously. The FWHM values obtained from the fitted W(110) profiles were used to calculate the apparent coherent diffraction domain size according to Equation (2).

The apparent coherent diffraction domain size shows an overall increase with irradiation temperature for all three exposure histories (Figure 4). The values increase from approximately 48–55 nm at 700 °C to 66–76 nm at 900 °C, with the largest change occurring between 900 and 1100 °C.

At 1100 °C, the H_2_-only specimen exhibited an apparent coherent diffraction domain size of approximately 118 nm, whereas the He-only and He → H_2_ specimens exhibited values near 103 nm. Thus, the He-containing conditions retain smaller apparent coherent diffraction domain sizes than the H_2_-only condition at this temperature. Because the instrumental contribution is not removed from the fitted peak widths, the absolute Scherrer-derived values may be systematically shifted; therefore, the difference observed at 1100 °C is interpreted primarily as a comparative difference in W(110) peak broadening rather than as an exact difference in physical grain size.

By 1300 °C, the values converge to approximately 126 nm for all three irradiation histories, and the differences between the exposure conditions become minimal.

This temperature-dependent trend is consistent with the SEM observations, which show a transition from relatively fine surface and fracture features at 700–900 °C to coarser grain-scale morphology at 1100–1300 °C.

The XRD results support the temperature-dependent structural trend observed by SEM. The increase in apparent coherent diffraction domain size with temperature is accompanied by the transition toward coarser grain-scale surface and fracture features. Thermally activated structural evolution is expected to contribute to this temperature-dependent increase in apparent diffraction-domain size. However, because the H_2_-only, He-only, and He → H_2_ specimens were compared at the same nominal irradiation temperatures, the differences observed among the exposure histories at a given temperature are consistent with an additional irradiation-history-dependent component superimposed on the common thermal evolution. This is particularly evident at 1100 °C, where the He-containing specimens show both a different W(110) diffraction response and more developed pore-like and cavity-like surface features than the H_2_-only specimen. The difference observed at 1100 °C is consistent with a different diffraction response of the He-containing specimens compared with the H_2_-only specimen. The ±15 °C pyrometer uncertainty is small relative to the 200 °C interval between the investigated temperature conditions and is not expected to alter the qualitative interpretation of the trends observed around the nominal 1100 °C condition. Temperature-dependent changes in the crystalline and mechanical response of He-irradiated tungsten have also been reported in previous plasma-exposure studies [22,23]. By 1300 °C, the convergence of the D values is consistent with the reduced morphological differences among the three exposure histories. This convergence suggests that thermally activated structural evolution becomes increasingly important at the highest investigated temperature. Because the Scherrer-derived values represent diffraction-coherent domains rather than directly measured grains, this convergence should not be interpreted as quantitative evidence of complete recrystallization.

Instrumented indentation was subsequently used to evaluate the nanomechanical response of tungsten after H_2_-only, He-only, and sequential He → H_2_ plasma exposure. Figure 5 presents the converted Vickers hardness HV and indentation modulus EIT as functions of irradiation temperature.

The converted Vickers hardness decreases with increasing irradiation temperature for all three exposure conditions (Figure 5a). At 700 °C, the mean hardness values are approximately 580 HV for H_2_-only, 645 HV for He-only, and 670 HV for He → H_2_. At 900 °C, the corresponding values are approximately 535, 610, and 545 HV, respectively. A further decrease is observed at 1100 °C, where the values are approximately 480–535 HV, followed by values of approximately 460–495 HV at 1300 °C.

The differences among the exposure histories are most evident at 700–900 °C. At 700 °C, the highest mean hardness is measured for the He → H_2_ specimen, whereas at 900 °C the He-only specimen exhibits the highest value. At 1100 and 1300 °C, the differences among the three conditions decrease, and the error-bar ranges partially overlap.

The indentation modulus EIT also decreases with increasing irradiation temperature (Figure 5b). For the H_2_-only series, EIT decreases from approximately 495 GPa at 700 °C to approximately 315 GPa at 1300 °C. The He-only series decreases from approximately 480 to 305 GPa, while the He → H_2_ series decreases from approximately 450 to 270 GPa over the same temperature range. The largest decrease occurs between 900 and 1100 °C for all three exposure conditions.

The He → H_2_ specimens exhibit the lowest mean EIT values throughout most of the investigated temperature range, particularly at 1100 and 1300 °C. In contrast to the hardness values, which become closer among the three exposure conditions at 1300 °C, differences in the indentation modulus remain visible at the highest temperature.

Overall, the indentation measurements show that both HV and EIT decrease with increasing irradiation temperature, while the magnitude of the differences among the H_2_-only, He-only, and He → H_2_ conditions depends on the measured mechanical parameter.

The nanomechanical response is consistent with a combined influence of irradiation-induced defects and temperature-dependent structural evolution. Previous studies have shown that He-related defect populations can modify the hardness of tungsten through interactions with vacancies and dislocations [20,21], while He-plasma exposure can produce temperature-dependent changes in both microstructure and mechanical properties [22]. In the present study, the larger hardness differences among the exposure histories at 700–900 °C indicate that the mechanical response remains sensitive to the irradiation condition in this temperature range. At 1100–1300 °C, the hardness values become closer, in parallel with the convergence of the apparent coherent diffraction domain size and the development of coarser grain-scale morphology. The indentation modulus, however, remains more strongly separated among the exposure conditions, particularly for the sequential He → H_2_ specimens. Because the measurements were performed at a maximum load of 5 mN, the measured HV and EIT values represent an effective near-surface mechanical response that may include contributions from both the plasma-modified region and the underlying tungsten. Accordingly, the indentation data are used comparatively among the irradiation conditions rather than to assign intrinsic mechanical properties to the modified layer alone.

Thermal desorption spectroscopy was used to compare hydrogen release from the H_2_-only and sequential He → H_2_ specimens irradiated at 700, 900, 1100, and 1300 °C. The unirradiated tungsten was included as a reference. During the TDS cycle, the specimens were heated from 100 to 1100 °C at 5 °C min−1 and then held at 1100 °C for 30 min. The corresponding temperature program is shown in Figure 6.

For both irradiation histories, the H_2_ signal remains relatively low during the initial and intermediate stages of the TDS cycle and increases during the high-temperature part of the heating program. The largest changes in signal occur near the end of the linear-heating stage and during the subsequent 1100 °C isothermal hold.

Based on the applied heating rate of 5 °C min^−1^, the onset of the sustained high-temperature H_2_ desorption increase was estimated from the TDS curves. For the H_2_-only specimens irradiated at 700, 900, 1100, and 1300 °C, the corresponding onset temperatures were approximately 850, 825, 800, and 775 °C, respectively. For the sequential He → H_2_ specimens, the corresponding onset temperatures were approximately 850, 825, 800, and 700–725 °C, respectively. These values represent approximate visual estimates of the beginning of the sustained desorption increase and exclude the short initial transient observed at the beginning of the TDS cycle.

For the H_2_-only series (Figure 6a), the 700 and 900 °C specimens exhibit similar high-temperature desorption profiles. The 1100 °C specimen shows a higher signal, while the 1300 °C specimen exhibits the highest response within this series. Its H_2_ signal reaches a maximum near the beginning of the 1100 °C hold and then decreases during the isothermal stage. The unirradiated tungsten shows the lowest high-temperature signal. In this regard, the TDS results are discussed primarily in terms of relative differences in desorption behavior across the irradiation regimes investigated.

Accordingly, differences in TDS signal intensity are interpreted on a comparative basis and are not expressed here as absolute values of the amount of retained hydrogen.

For the sequential He → H_2_ series (Figure 6b), the 700 and 900 °C specimens again show comparable desorption profiles. The 1100 °C specimen develops a distinct maximum shortly after the beginning of the isothermal hold, followed by a decrease in signal. The 1300 °C specimen exhibits the highest H_2_ response of the sequential series and maintains an elevated signal over most of the 30 min hold.

Comparison of the H_2_-only and He → H_2_ series shows that the differences between the two exposure histories depend on irradiation temperature. The differences between the two exposure histories are smaller at 700 and 900 °C and become more evident at 1100 and 1300 °C. At 1100 °C, the sequentially irradiated specimen exhibits a more pronounced high-temperature maximum, whereas at 1300 °C the He → H_2_ specimen shows a prolonged high-temperature desorption response during the isothermal hold.

Under the present sequential protocol, the He → H_2_ condition is associated with differences in both the magnitude and temporal evolution of the H_2_ desorption signal. The stronger high-temperature desorption observed for the sequential He → H_2_ specimens is consistent with hydrogen release from relatively stable trapping sites formed or modified during the preceding He exposure. Candidate sites include He–vacancy complexes, vacancy clusters, and cavity-associated open-volume defects in the near-surface region. The SEM observations provide complementary morphological context through the more developed pore-like and cavity-like features observed in the He-containing specimens, particularly at 1100 °C. Accordingly, the assignment of specific trapping sites is treated as a mechanistic interpretation based on the combined SEM and TDS response and on previously reported He–H interactions in tungsten [13,14,15,16,17,18,19].

The temperature-dependent differences observed for the sequential He → H_2_ condition are consistent with previous sequential He–H/D studies. Nishijima et al. showed that He pre-exposure can either decrease or increase subsequent deuterium retention depending on irradiation temperature [17]. Xia et al. further demonstrated that a pre-existing He-modified layer can alter subsequent hydrogen transport and surface evolution during sequential exposure [18]. More recent work has also shown that suppression of visible surface damage after He pre-exposure does not necessarily correspond to reduced hydrogen-isotope retention [19]. The present results follow the same general trend: the differences between the H_2_-only and sequential He → H_2_ exposure histories are smaller at 700–900 °C but become more evident in the high-temperature desorption behavior at 1100 and 1300 °C.

At 1100 °C, the present results can also be qualitatively compared with our previous mixed He/H_2_ plasma study [25]. Under simultaneous He/H_2_ exposure, increasing the He fraction produced a progressively more developed porous near-surface morphology and modified the high-temperature hydrogen-desorption response. In the present sequential experiment, the He-containing specimens similarly exhibit more developed pore-like and cavity-like surface features at 1100 °C, while the He → H_2_ specimen shows a distinct high-temperature H_2_ desorption maximum relative to the H_2_-only condition. Thus, both mixed and sequential He/H_2_ exposure show an association between He-induced modification of the near-surface defect structure and subsequent hydrogen-release behavior. The present sequential data show a similar association when the He-modified surface is established before the H_2_-plasma stage.

Comparison of all four characterization methods shows that the irradiation-history effect is not expressed identically in structural, mechanical, and hydrogen-desorption measurements. The sequential results are therefore interpreted in the context of the complete two-stage He → H_2_ irradiation history, which includes the preceding He exposure, the longer total exposure duration, and the corresponding stage-specific fluences, rather than as an isolated gas-ordering effect. At 1100 °C, the He-containing specimens exhibit more developed pore-like and cavity-like surface features and smaller apparent coherent diffraction domain sizes than the H_2_-only specimen. At the same irradiation temperature, the sequential He → H_2_ specimen exhibits a distinct high-temperature H_2_ desorption maximum. These observations indicate that the structural state produced by prior He exposure is associated with a modified subsequent hydrogen-release response. At 1300 °C, the surface morphology, hardness, and apparent coherent diffraction domain size become more similar among the three exposure histories, whereas the TDS profiles remain different. Thus, convergence of the observable structural and mechanical characteristics does not necessarily imply identical hydrogen trapping and release behavior. Each temperature–exposure condition was represented by one independently irradiated specimen. Accordingly, the reported nanoindentation uncertainty reflects spatial variation within each specimen, while comparisons among the irradiation conditions are interpreted primarily in terms of the overall temperature-dependent and irradiation-history-dependent trends. The irradiation-history effects identified here are specific to the plasma fluxes, fluences, temperatures, and exposure sequence investigated in the KAZ-PSI experiments. Nevertheless, the observed association between He-induced near-surface modification and subsequent hydrogen-desorption behavior is also consistent with our previous mixed He/H_2_ plasma study at 1100 °C [25]. Together, these results suggest that irradiation history and He-modified defect structures remain relevant parameters under both sequential and mixed-species exposure, although the magnitude of the response may vary with plasma flux, fluence, species composition, and exposure duration.

## 4. Conclusions

Sequential He → H_2_ plasma exposure produces a temperature-dependent modification of the surface morphology, crystalline structure, nanomechanical response, and hydrogen desorption behavior of tungsten.

The surface morphology changes with both irradiation temperature and exposure history. At 700 °C, the initial polishing relief remains visible for all three exposure conditions. The largest morphological differences between the H_2_-only, He-only, and He → H_2_ specimens are observed at 1100 °C, where the He-containing specimens exhibit a more developed pore-like and cavity-like surface morphology. At 1300 °C, all three conditions show predominantly grain-scale surface morphology, and the differences among the exposure histories become smaller. The gas composition of the observed pore-like features cannot be determined from SEM alone.

XRD confirms preservation of the bcc-W structure under all investigated conditions, with no detectable secondary crystalline phases. The apparent coherent diffraction domain size increases from approximately 48–55 nm at 700 °C to approximately 126 nm at 1300 °C. At 1100 °C, the H_2_-only specimen reaches approximately 118 nm, whereas the He-only and He → H_2_ specimens remain near 103 nm. At 1300 °C, the values converge for all three exposure histories.

The nanomechanical response also varies systematically with irradiation temperature. Both the converted Vickers hardness and indentation modulus decrease with increasing temperature. The converted Vickers hardness shows the differences among the exposure histories at 700–900 °C and becomes more similar at 1100–1300 °C. The indentation modulus EIT decreases from approximately 450–495 GPa at 700 °C to approximately 270–315 GPa at 1300 °C, with the He → H_2_ specimens generally exhibiting the lowest mean values at the higher temperatures.

TDS shows that the H_2_ desorption response varies with irradiation temperature and exposure history. The main hydrogen release occurs during the high-temperature part of the TDS cycle and during the 1100 °C isothermal hold. Differences between the H_2_-only and He → H_2_ conditions are relatively small at 700–900 °C and become more evident at 1100 and 1300 °C. At 1100 °C, the He → H_2_ specimen exhibits a distinct high-temperature desorption maximum, whereas at 1300 °C an elevated H_2_ signal persists over a larger part of the 30 min isothermal hold. Under the present sequential protocol, the He → H_2_ specimens exhibit differences in both the magnitude and temporal evolution of hydrogen release.

Overall, the response associated with the sequential He → H_2_ exposure history varies across the investigated temperature range. Differences in surface morphology, apparent coherent diffraction domain size, and nanomechanical properties are most evident in the intermediate-temperature range and decrease at 1300 °C, whereas differences in hydrogen desorption remain detectable at the two highest irradiation temperatures. Thus, convergence of the structural and mechanical characteristics at 1300 °C does not necessarily correspond to identical hydrogen-release behavior.

## Figures and Tables

**Figure 1:**
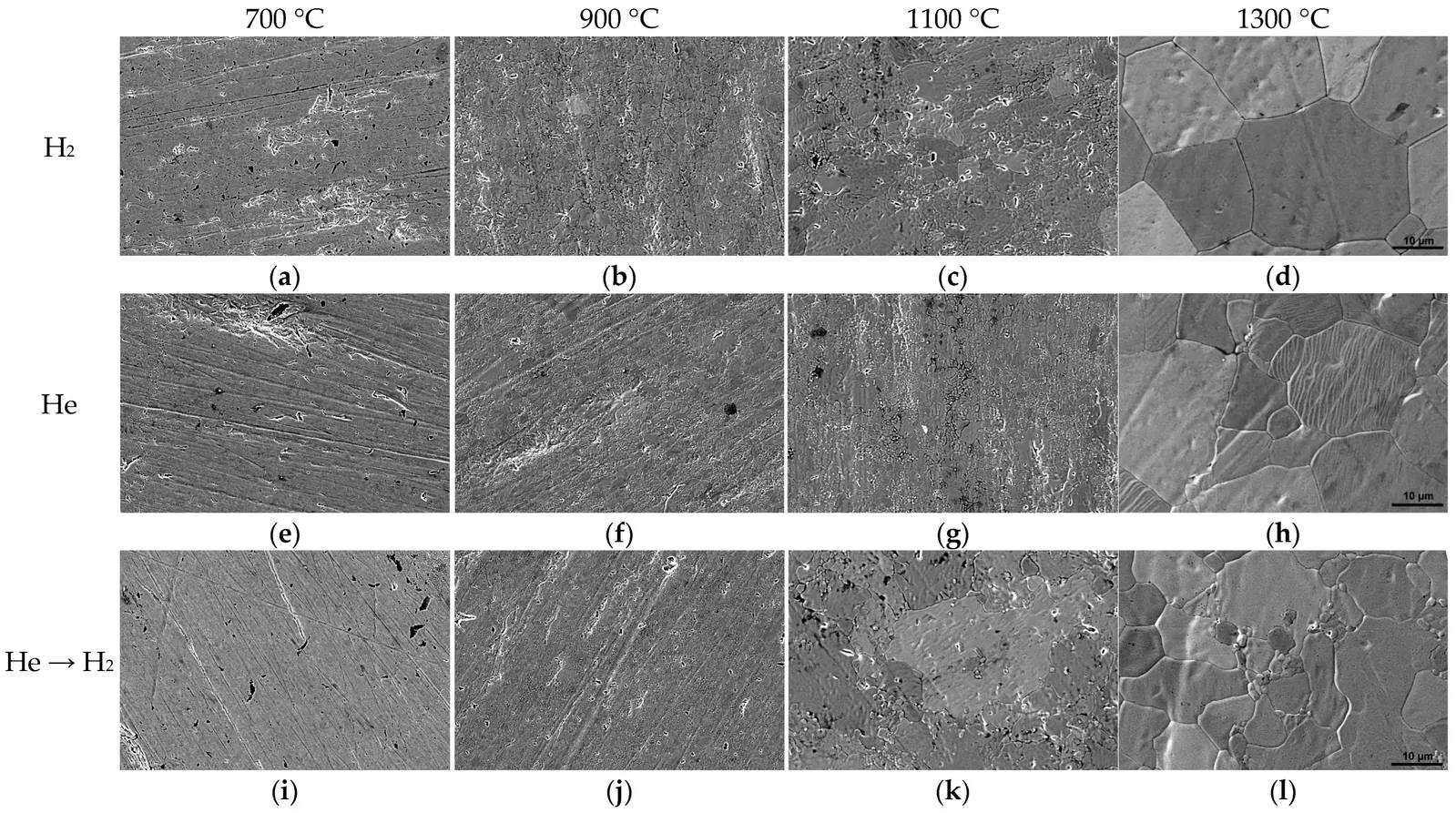
SEM images of tungsten surfaces after plasma exposure: (**a**–**d**) H_2_-only, (**e**–**h**) He-only, and (**i**–**l**) sequential He → H_2_ exposure at 700, 900, 1100, and 1300 °C, respectively.

**Figure 2:**
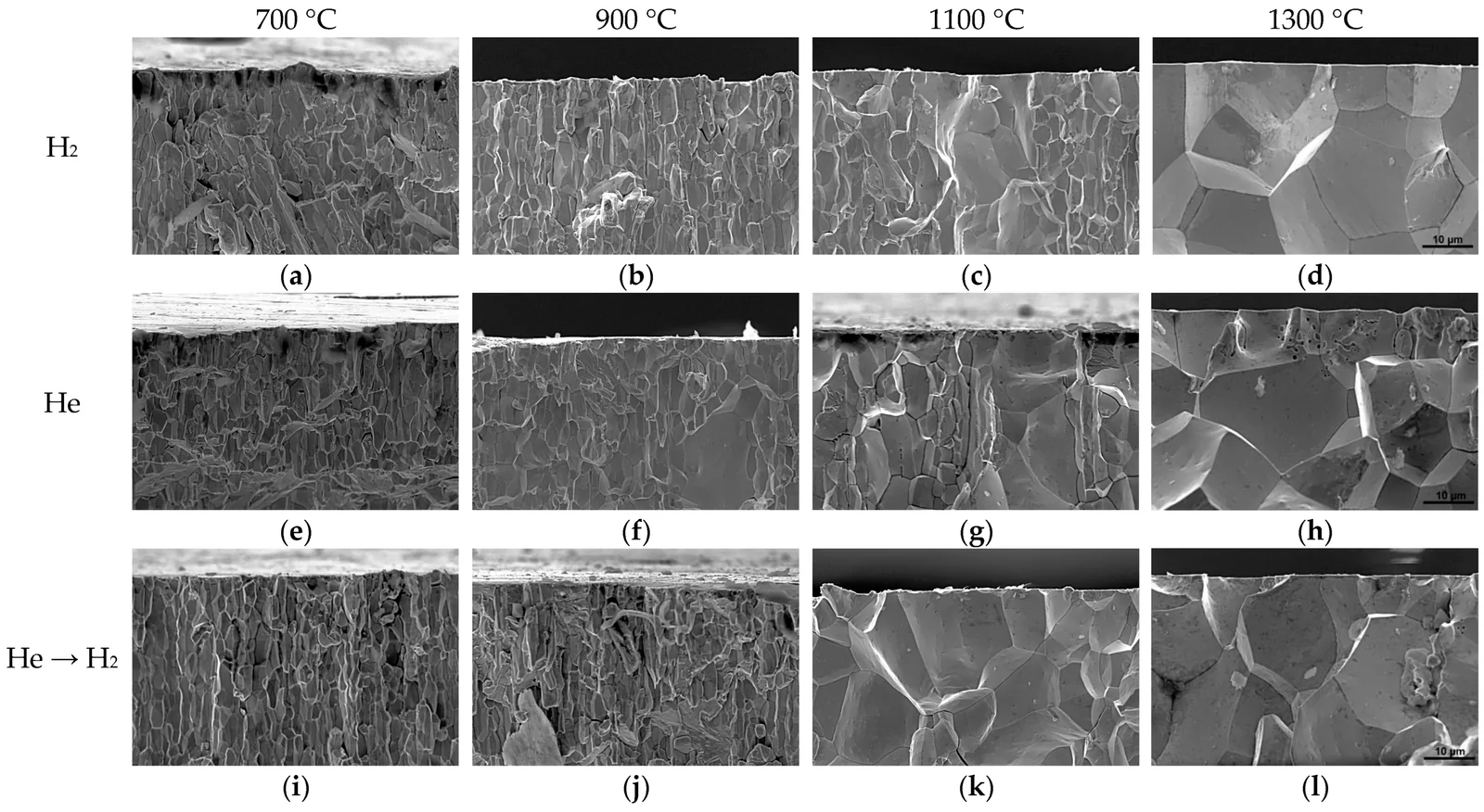
Cross-sectional fracture SEM images of tungsten after plasma exposure: (**a**–**d**) H_2_-only, (**e**–**h**) He-only, and (**i**–**l**) sequential He → H_2_ exposure at 700, 900, 1100, and 1300 °C, respectively.

**Figure 3:**
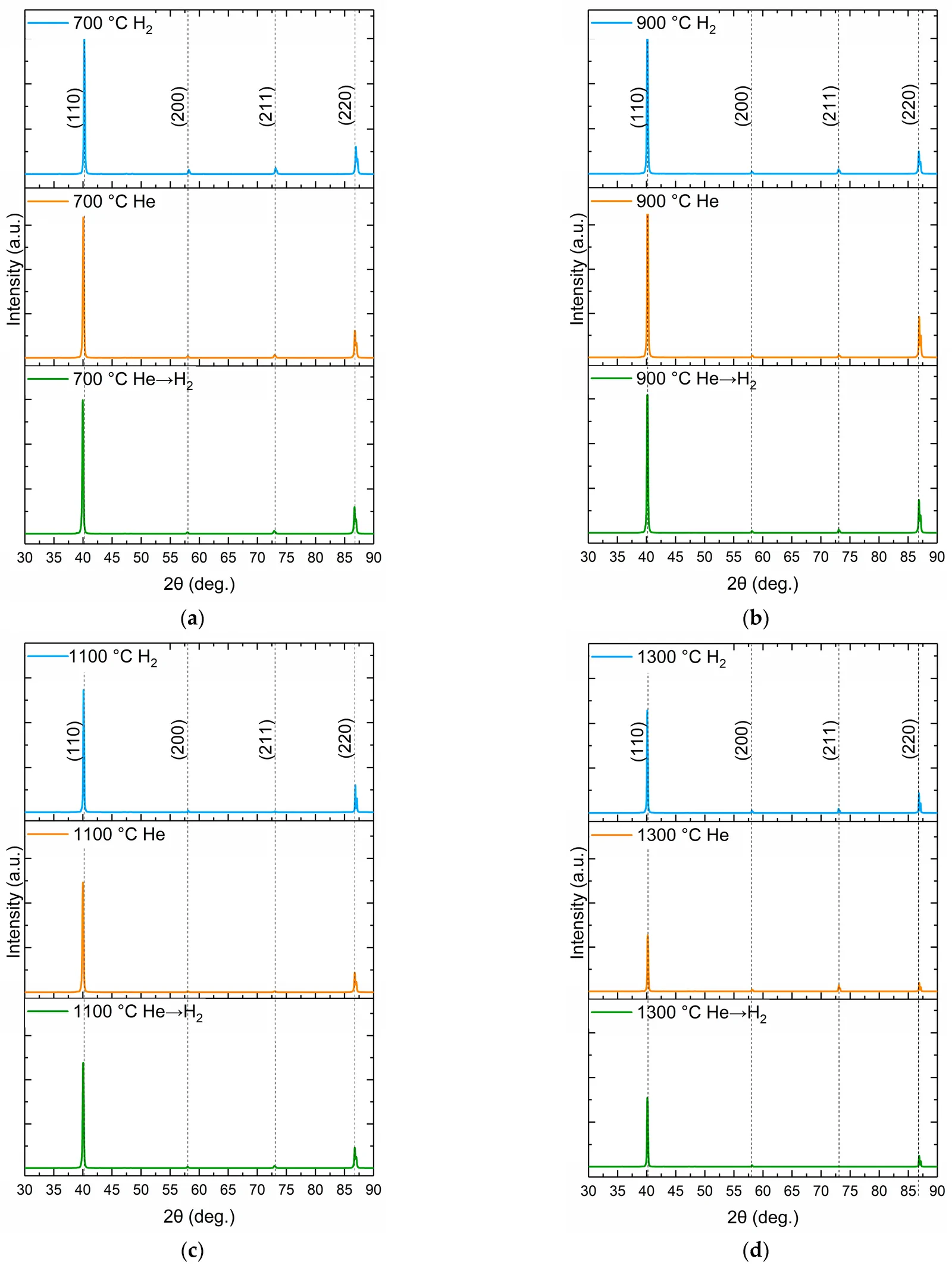
XRD patterns of tungsten after H_2_-only, He-only, and sequential He → H_2_ plasma exposure at (**a**) 700 °C, (**b**) 900 °C, (**c**) 1100 °C, and (**d**) 1300 °C. The principal reflections correspond to bcc-W (110), (200), (211), and (220). The patterns are presented separately within each panel for clarity.

**Figure 4:**
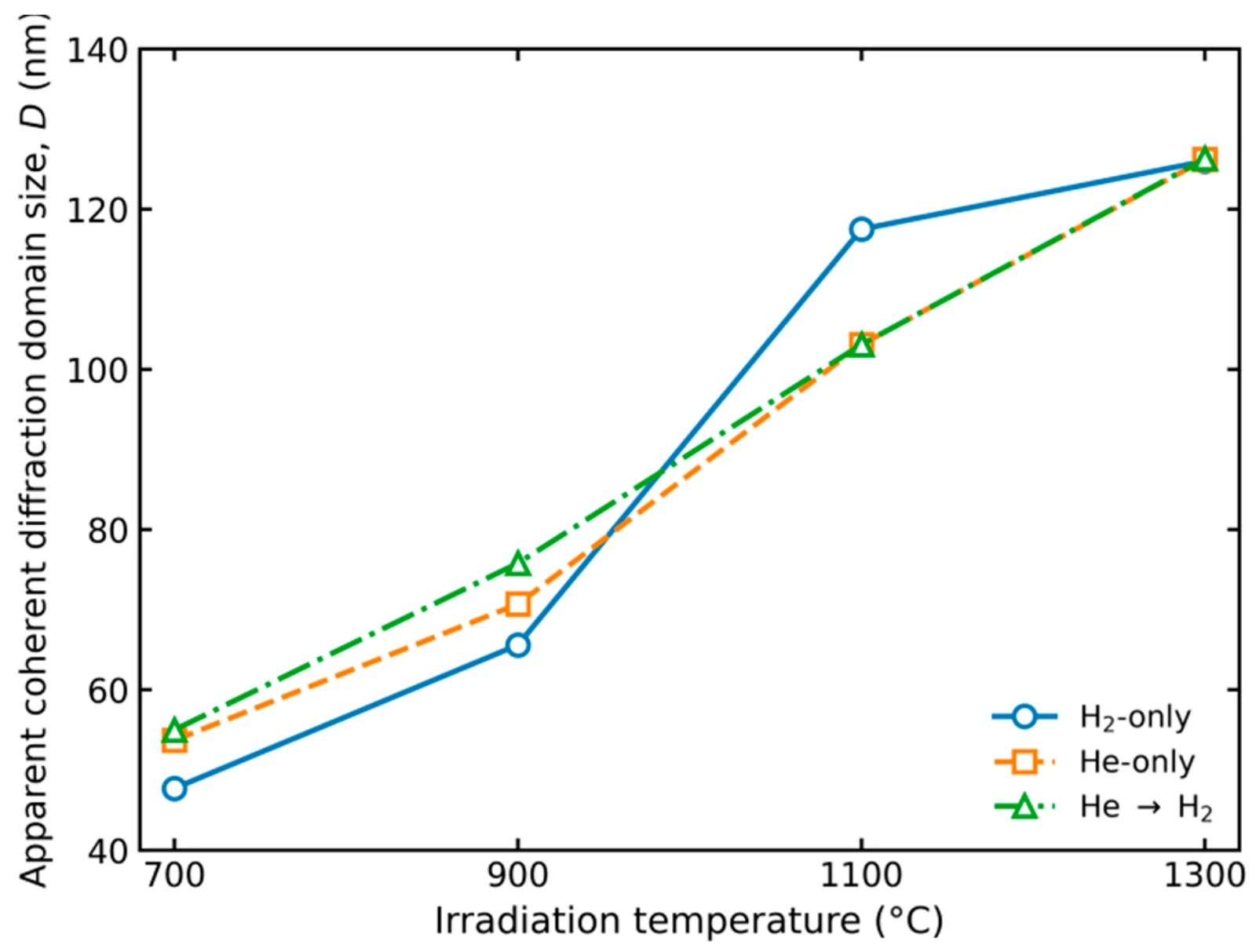
Apparent coherent diffraction domain size of tungsten calculated from the W(110) reflection as a function of irradiation temperature for H_2_-only, He-only, and sequential He → H_2_ plasma exposure.

**Figure 5:**
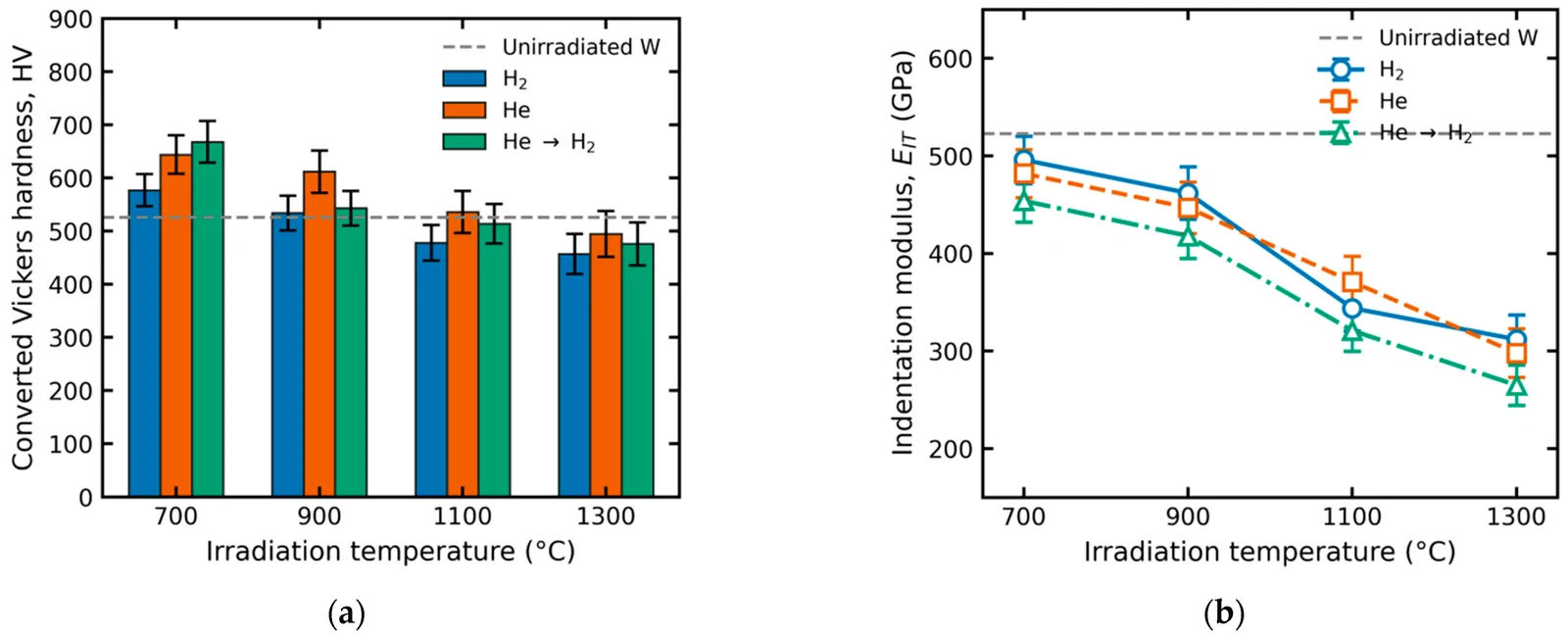
Temperature dependence of the nanomechanical properties of tungsten after H_2_-only, He-only, and sequential He → H_2_ plasma exposure: (**a**) converted Vickers hardness (HV) and (**b**) indentation modulus (EIT). Error bars represent the standard deviation of valid indentation measurements. The dashed line and shaded region indicate the mean value and standard deviation of the unirradiated tungsten, respectively.

**Figure 6:**
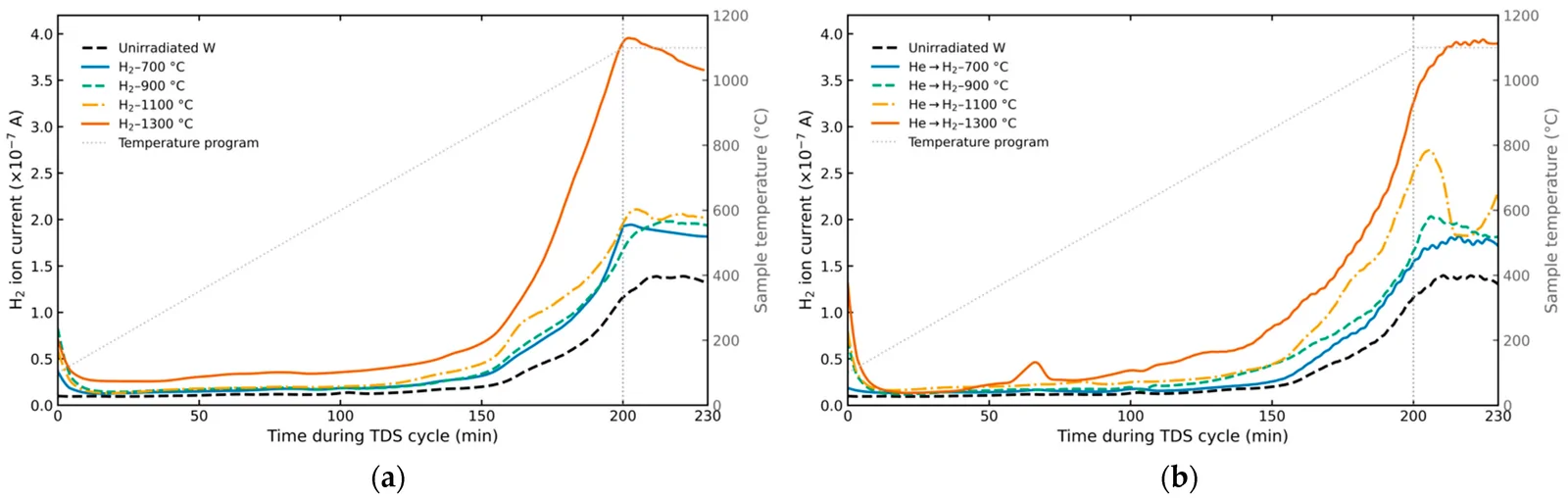
Thermal desorption behavior of tungsten after (**a**) H_2_-only plasma exposure and (**b**) sequential He → H_2_ plasma exposure at 700, 900, 1100, and 1300 °C. The H_2_ signal was monitored at *m*/*z* = 2. The grey dotted line represents the temperature program. The specimens were heated from 100 to 1100 °C at 5 °C min^−1^ and subsequently held at 1100 °C for 30 min. The unirradiated tungsten is shown as a reference.

**Table 1:** Plasma exposure conditions for the He-only, H_2_-only, and sequential He → H_2_ irradiation experiments.

Parameter	He-Only	H_2_-Only	He → H_2_
Exposure temperature, °C	700, 900, 1100, 1300	700, 900, 1100, 1300	700, 900, 1100, 1300
He exposure time, h	1	—	1
H_2_ exposure time, h	—	1	1
Total exposure time, h	1	1	2
Gas flow rate, sccm	≈50 He	≈50 H_2_	≈50 He/≈50 H_2_
Working pressure, Pa	≈1	≈1	≈1
Estimated Ion flux, Γ, 10^22^ m^−2^ s^−1^	≈0.95± 0.14	≈1.45± 0.22	≈0.95 ± 0.14 (He stage); ≈1.45 ± 0.22 (H_2_ stage)
Calculated Fluence, Φ, 10^25^ m^−2^	≈3.42 ± 0.51	≈5.22 ± 0.78	≈3.42 ± 0.51 (He stage); ≈5.22 ± 0.78 (H_2_ stage)

Note: “—” indicates not applicable.

## Data Availability

The original contributions presented in this study are included in the article. Further inquiries can be directed to the corresponding author.
